# Intra-pancreatic release of bradykinin during the course experimental pancreatitis in rat

**DOI:** 10.1155/S0962935196000282

**Published:** 1996-06

**Authors:** P. Jönsson, K. Ohlsson

**Affiliations:** 1 Department of Surgical Pathophysiology University Hospital MAS Lund University Malmö Sweden

## Abstract

Kallikrein/Kininogn activation is an important pathophysiological event in acute pancreatitis, leading to microcirculatory changes within the gland. Hitherto, only indirect measurements of pancreatic bradykinin formation have been performed, monitoring the peptide in the circulation and in the peritoneal exudate. In the present study, intra-pancreatic bradykinin release was assessed using microdialysis during experimental acute pancreatitis in rat. In mild, oedematous pancreatitis, induced by caerulein hyperstimulation, the levels of bradykinin within the gland were not elevated compared with those of control rats. However, in necrotic pancreatitis, induced by retrograde injection of taurocholate into the pancreatic duct, significantly elevated levels of intraglandular bradykinin were seen. Several rats in this group died whilst in a state of circulatory shock.


Mediators of Inflammation, 5, 202–205 (1996)

### Intra-pancreatic release of bradykinin during the course of experimental pancreatitis in rat

P. Jönsson[CA] and K. Ohlsson

Department of Surgical Pathophysiology, University Hospital MAS, Lund University, Malmö, Sweden

[CA]Corresponding Author

**KALLIKREIN/KININOGEN activation is an important pathophysiological event in acute pancreatitis, leading to microcirculatory changes within the gland. Hitherto, only indirect measurements of pancreatic bradykinin formation have been performed, monitoring the peptide in the circulation and in the peritoneal exudate. In the present study, intra-pancreatic bradykinin release was assessed using microdialysis during experimental acute pancreatitis in rat. In mild, oedematous pancreatitis, induced by caerulein hyperstimulation, the levels of bradykinin within the gland were not elevated compared with those of control rats. However, in necrotic pancreatitis, induced by retrograde injection of taurocholate into the pancreatic duct, significantly elevated levels of intraglandular bradykinin were seen. Several rats in this group died whilst in a state of circulatory shock.**

**Key words:** Bradykinin, Caerulein, Microdialysis, Pancreatitis, Rat, Taurocholate

### Introduction

Release of bradykinin has been demonstrated during acute pancreatitis and is proposed to contribute to the development of hypotension and shock.[1,2] Bradykinin is one of the most potent vasodilators in man, causing loss of intravascular fluid by increasing the capillary permeability.[3] It may also generate severe pain. Bradykinin is formed in blood through limited proteolysis of the kininogens by activated plasma kallikrein. Kallidin (lysyl-bradykinin) is released from kininogens by glandular kallikrein and may be converted to bradykinin by amino peptidases in plasma.[4] In pancreatitis, trypsinogen is activated to trypsin in the pancreatic gland.[5,6] Trypsin may then activate the pro-kallikreins and also directly release bradykinin from kininogen.[2]

Direct measurement of activated bradykinin in the circulation is difficult due to the great capacity of blood to both generate and degrade this peptide.[7,8] The kinins are rapidly inactivated by carboxypeptidases (kinase I and II) and have an estimated half-life of about 20 s in the human circulation.[9] The plasma levels of bradykinin reported for man vary considerably when measured by bio-assay and radioimmunoassay.[10,11]

The microdialysis technique[12–15] offers the possibility of studying the bradykinin release directly in the interstitial fluid of the pancreas, by selecting probe membranes with a permeability allowing the passage of bradykinin (MW ≈ 1 kDa) but not of the kinases (MW ≈ 45 kDa). The purpose of this work was to elucidate whether the release of bradykinin in the pancreatic gland is more profuse during taurocholate-induced pancreatitis in rat, often leading to shock, than during caerulein-induced pancreatitis.

### Materials and Methods

*Animal experiments:* Adult Wistar rats of both sexes, weighing 205–260 g were obtained from Möllegaard Avelslab A/S (Skensved, Denmark). The animals were deprived of food but not of water for 18 h before the experiments. Anaesthesia was maintained by repeated injections of mebumal (barbital) into the peritoneal cavity. The body temperature was continuously monitored and adjusted if necessary. A polyethylene catheter (PE 90) was introduced into a femoral vein. All animals received saline intravenously at 4 ml/h. The pancreas was exposed through a mildline incision. Rats were randomly segregated into three groups; caerulein-induced pancreatitis ($n = 6$), taurocholate-induced pancreatitis ($n = 8$) and controls ($n = 6$).

Caerulein pancreatitis was induced by intravenous infusion of caerulein (Sigma, St Louis, MO, USA), 10 µg/kg/h for 6 h.

Taurocholate pancreatitis was induced by retrograde injection of sodium taurocholate (Sigma, St Louis, MO, USA) into the pancreatic duct system. The common bile and pancreatic duct was cannulated trans-duodenally and the duct was temporarily occluded proximal of the con-

© 1996 Rapid Science Publishers necting pancreatic ductuli by application of a vascular clamp. Taurocholate diluted in saline, 0.8 ml 4% (w/v), was injected into the common duct for 90 s and the clamp was removed.

All three groups were subjected to microdialysis of the extracellular fluid of the pancreatic gland. A 10 mm long probe, with a diameter of 0.5 mm, was introduced into the central part of the pancreatic gland. The polycarbonate membrane had a molecular weight cut-off of less than 20 kDa. A micro-injection pump (CMA/100) and a micro-sampler (CMA/200) were used (CMA/Microdialysis AB, Stockholm, Sweden). Ringer's solution (Baxter, Norway) was used as perfusion fluid at a flow rate of 5 μl/min and 30 min fractions were collected at +4°C in the fraction collector and stored at −70°C until analysed. Microdialysis was started when pancreatitis was induced.

Samples of venous blood were drawn at 0 h, 2 h and 6 h into EDTA-containing tubes. After centrifugation, the plasma samples were stored at −20°C until analysed.

All animals surviving the observation period were killed with an overdose of mebumal. The study was approved by the ethics committee of Lund University.

*Assays:* Bradykinin was measured using a radioimmunoassay technique described previously.[10] Briefly, $^{125}$I-tyr$^{8}$-bradykinin was obtained from NEN (Du Pont, Wilmington, DE, USA) and used as tracer. The specific activity was 1 800 μCi/μg. The tracer was reconstituted with distilled water, diluted to 50 μCi/ml and kept at −70°C. The RIA buffer included barbital 0.075 M and bovine serum albumin 0.2% at pH 8.6. A 0.2 ml sample or standard was incubated with tracer, 0.05 ml (30 000 cpm), and antibody solution, 0.05 ml (1/2 500) in the RIA buffer. The reaction mixture was incubated at +4°C for 18 h. For the separation, 1 ml of the buffer containing 0.15% charcoal and 0.015% dextran P70 was added. After centrifugation at 2 500 × **g** for 15 min, 1 ml of the supernatant was transferred to a disposable plastic tube and counted for 20 min in a gamma scintillation counter. The sensitivity of the RIA, defined as the lowest standard producing a significant displacement of labelled antigen, was 10 μg/l. With the bradykinin cross-reaction set at 100%, the antiserum cross-reacted with kallidin to 97%, with metionyl-lysyl bradykinin to 42% and with des-arginine-bradykinin to 82%. Amylase was measured using the Phadebas Amylase Test (Pharmacia Diagnostica AB, Uppsala, Sweden) and $\alpha_2$-acute phase protein ($\alpha_2$-AP) was measured in the blood samples using electroimmunoassay.[16]

*Statistics:* Results are expressed as means (±S.E.M.). The Mann–Whitney *U*-test was used to determine possible differences between groups of rats with pancreatitis and the control group. A *p* value less than 0.05 was considered significant.

### Results

All animals in the control group and in the caerulein group survived the observation period of 6 h, while three of the animals in the taurocholate group died. At autopsy, a severe inflammatory reaction was seen in the pancreatic gland in the taurocholate group with haemorrhage, oedema and necrotic areas, while in the caerulein group, only modest oedema was seen, without bleeding or obvious necrosis. The microdialysis procedure did not cause any significant inflammatory reaction. The microscopic appearance of the pancreas was essentially normal after 6 h of microdialysis (Fig. 1).

The concentration of bradykinin in the microdialysate from taurocholate rats showed a rapid

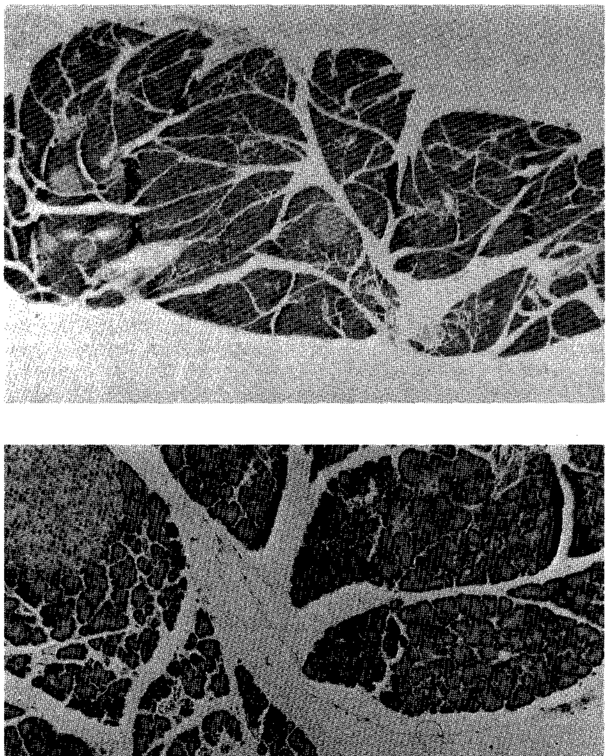

FIG. 1. Rat pancreas stained in haematoxylin–eosin after 6 h of microdialysis (×30 and ×125). Apart from slight interlobular oedema, the microscopic appearance of acini and lobuli was normal. No infiltration of inflammatory cells was seen.

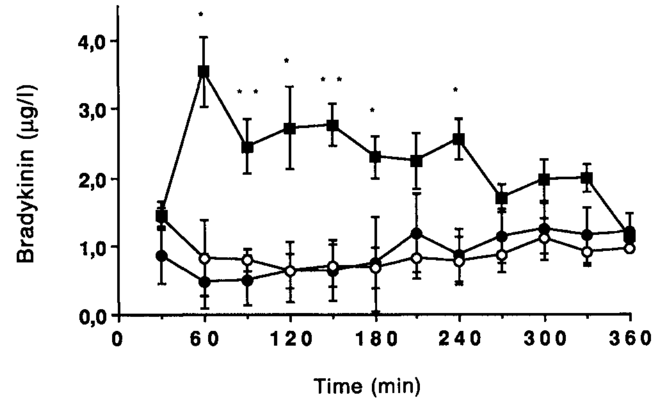

FIG. 2. Dialysate levels, mean (±S.E.M.), of immunoreactive bradykinin during taurocholate- (■) and caerulein- (●) induced pancreatitis in rats, compared with controls (○). *$p < 0.05$ and **$p < 0.01$ indicate differences between control and taurocholate rats.

increase, with a mean maximum value of 3.5 µg/l 1 hour after induction of the pancreatitis (Fig. 2). The level was significantly increased for about 4 h compared with the control rats. The increase in bradykinin concentration in the dialysates from the animals with caerulein-induced pancreatitis was modest and did not differ significantly from that of the control group.

The caerulein group showed the most pronounced increase in amylase levels with a mean value of 450 µcat/l after 6 h. In comparison, the mean value of the taurocholate group was 180 µcat/l and the control group 70 µcat/l after 6 h (Fig. 3).

The plasma concentration of $\alpha_2$-AP increased in all three groups, but the most pronounced increase was seen in the taurocholate group (Fig. 4).

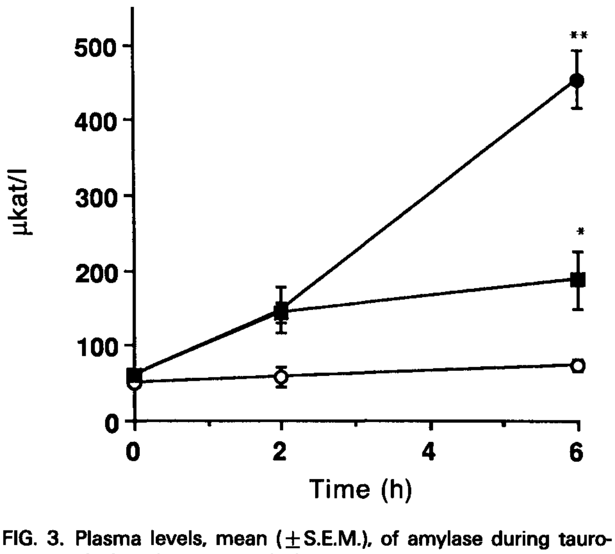

FIG. 3. Plasma levels, mean (±S.E.M.), of amylase during taurocholate- (■) and caerulein- (●) induced pancreatitis in rats and amylase levels in the control group (○). *$p < 0.05$ and **$p < 0.01$ indicate differences between control rats and rats with pancreatitis.

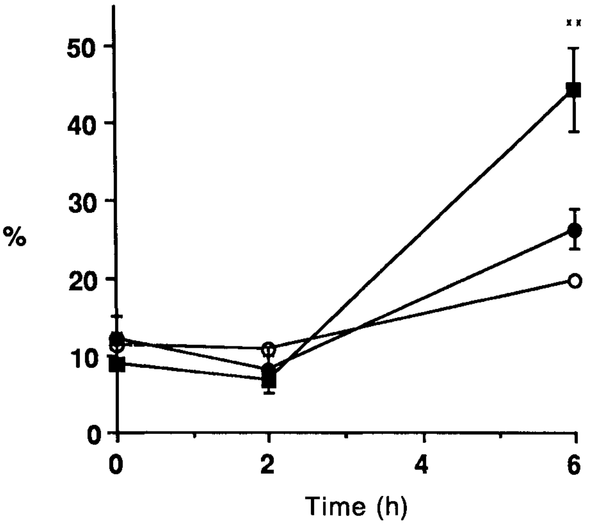

FIG. 4. Plasma levels, mean (±S.E.M.), of $\alpha_2$-AP in rats during taurocholate- (■) and caerulein- (●) induced pancreatitis and in control animals (○). *$p < 0.05$ and **$p < 0.01$ indicate differences between control rats and rats with pancreatitis.

### Discussion

The activation of kininogen is an important pathophysiological event in acute pancreatitis. Bradykinin antagonists may improve the outcome in experimental pancreatitis by reducing pancreatic oedema and hypotension.[17,18] Measurements of activated bradykinin have been performed in blood and peritoneal exudate, but not where the inflammatory process begins—in the pancreas. We have earlier adapted the microdialysis technique for the exocrine pancreas.[13,14] In this study we utilized the system for the evaluation of intrapancreatic bradykinin release during experimental pancreatitis. Introduction of the microdialysis probe into the pancreatic gland caused only a minor trauma to the tissue, as judged from the low initial bradykinin level in the dialysate. Furthermore, the microscopic appearance of the pancreatic tissue was normal after 6 h of microdialysis. Microdialysis has already been used to measure bradykinin in clinical studies, not concerned with pancreas, but after oral surgery.[15,19] The membrane of the probe does not induce bradykinin formation.[15]

In this study, we used two different models of experimental pancreatitis. Hyperstimulation of the pancreatic gland with caerulein, a synthetic cholecystokinin analogue, produces a mild oedematous form of pancreatitis in 1–2 h which later restitutes.[20,21] This form of pancreatitis did not cause release of bradykinin into the interstitium of the pancreatic gland. Increased levels of bradykinin have recently been demonstrated in venous blood from rats with caerulein-induced pancreatitis.[22] Differences in methodology and types of test samples prelude detailed comparisons, but our results indicate that the bradykinin release in this type of pancreatitis is rather low, at least in comparison with the taurocholate model. On the other hand, the highly elevated serum amylase levels reflect hyperstimulation of the pancreas.

Injection of taurocholate into the pancreatic duct induces severe pancreatitis, as evidenced by the autopsy findings and the mortality in this group during the observation period. This form of experimental pancreatitis is connected with grave microcirculatory disturbances in the pancreatic gland and, eventually, hypotension and shock.[23,24] The severity of the disease is also illustrated by the pronounced release of bradykinin within the gland, as evidenced by the high levels in the dialysate. These results are also in agreement with earlier, more indirect, findings indicating that the kinin release in acute pancreatitis was localized to the abdominal region, as illustrated by kininogen consumption in the peritoneal exudates.[2] The very rapid acute phase protein $\alpha_2$-AP showed much higher plasma levels in the taurocholate group than in the caerulein group and in the controls, reflecting the more severe inflammation in the taurocholate group.

In conclusion, microdialysis appears to offer a convenient method of monitoring the intra-pancreatic release of bradykinin and should also be applicable to the study of other peptides. Severe pancreatitis appears to be accompanied by an extensive release of bradykinin within the gland, probably contributing to the oedema and tissue damage seen in this disease.

### References

1. Ofstad E. Formation and destruction of plasma kinins during experimental acute hemorrhagic pancreatitis in dogs. *Scand J Gastroenterol* 1970; **5**: 7–44.
2. Lasson Å, Ohlsson K. Changes in the kallikrein kinin system during acute pancreatitis in man. *Thrombosis Research* 1984; **35**: 27–41.
3. Fox RH, Goldsmith RJ, Lewis GP. Bradykinin as a vasodilator in man. *J Physiol* 1961; **157**: 589–602.
4. Oates JA, Pettinger WA, Doctor RB. Evidence for release of bradykinin in carcinoid syndrome. *J Clin Invest* 1996; **45**: 173–178.
5. Ohlsson K, Genell S. Role of enzyme inhibitors in acute pancreatitis and rationale for their therapeutic use. In: Braganza JM, ed. *The Pathogenesis of Pancreatitis.* Manchester: Manchester University Press, 1991; 198–214.
6. Willemer S, Adler G. Mechanism of acute pancreatitis. Cellular and subcellular events. *Int J Pancreatol* 1991; **9**: 21–30.
7. Webster ME, Pierce JV. The nature of the kallidins released from human plasma by kallikreins and other enzymes. *Ann NY Acad Sci* 1963; **104**: 91–107.
8. Erdos EG, Sloane EM. Enzyme in human blood plasma that inactivates bradykinins and kallidins. *Biochem Pharmacol* 1962; **11**: 585–592.
9. Ward PE. Metabolism of bradykinin and bradykinin analogues. In: Burch RM, ed. *Bradykinin Antagonists: Basic and Clinical Research (Inflammatory Diseases and Therapy).* New York: Marcel Dekker, 1991; 147–170, vol. 5).
10. Hulthén UL, Borge T. Determination of bradykinin in blood by a sensitive radioimmunoassay. *Scand J Clin Lab Invest* 1976; **36**: 833–839.
11. Bönner G, Iwersen D. The analytical value for kinin concentration in blood depends on the antiserum used in the bradykinin radioimmunoassay. *J Clin Chem Clin Biochem* 1987; **25**: 39–43.
12. Ungerstedt U. Microdialysis—principles and applications for studies in animals and man. *J Intern Med* 1991; **230**: 365–373.
13. Jönsson P, Börgström A, Ohlsson K. Measurements of exocrine proteins in the pig pancreas using microdialysis. *Gastroenterol Jpn* 1992; **27**: 529–535.
14. Jönsson P, Ohlsson K. Intrapancreatic turnover of recombinant human pancreatic secretory trypsin inhibitor in experimental porcine pancreatitis. *Scand J Clin Lab Invest* 1995; **55**: 223–227.
15. Hargreaves KM, Costello A. Glucocorticoids suppress levels of immunoreactive bradykinin in inflamed tissue as evaluated by microdialysis probes. *Clin Pharmacol Ther* 1990; **48**: 168–178.
16. Laurell CB. Electroimmunoassay. *Scand J Clin Lab Invest* 1972; **29**: 21–37.
17. Griesbacher T, Lembeck F. Effects of the bradykinin antagonist, Hoe 140, in experimental acute pancreatitis. *Br J Pharmacol* 1992; **107**: 356–360.
18. Griesbacher T, Tiran B, Lembeck F. Pathological events in experimental acute pancreatitis prevented by the bradykinin antagonist, Hoe 140. *Br J Pharmacol* 1993; **108**: 405–411.
19. Swift JQ, Garry MG, Roszkowski MT, Hargreaves KM. Effect of flurbiprofen on tissue levels of immunoreactive bradykinin and acute postoperative pain. *J Oral Maxillofac Surg* 1993; **51**: 112–116.
20. Lampel M, Kern HF. Acute interstitial pancreatitis in the rat induced by excessive doses of a pancreatic secretagogue. *Virchows Arch [A]* 1977; **373**: 97–117.
21. Adler G, Hupp T, Kern HF. Course and spontaneous regression of acute pancreatitis in the rat. *Virchows Arch [A]* 1979; **382**: 31–47.
22. Shimizu I, Wada S, Okahisa T, *et al.* Radioimmunoreactive plasma bradykinin levels and histological changes during the course of cerulein-induced pancreatitis in rats. *Pancreas* 1993; **8**: 220–225.
23. Aho HJ, Koskensalo SM-L, Nevalainen TJ. Experimental pancreatitis in the rat. Sodium taurocholate-induced acute haemorrhagic pancreatitis. *Scand J Gastroenterol* 1980; **15**: 411–416.
24. Kusterer K, Enghofer M, Zendler S, Blöchle C, Usadel KH. Microcirculatory changes in sodium taurocholate-induced pancreatitis in rats. *Am J Physiol* 1991; **260**: G346–G351.

ACKNOWLEDGEMENTS. This study was supported by grants from the Swedish Medical Research Council (03910), the Medical Faculty at the University of Lund, the Albert Påhlsson Foundation and the Foundations for Medical and Cancer Research administered by Malmö University Hospital.

**Received 24 January 1996; accepted 22 February 1996**
